# On the Intensity of the Microvascular Magnetic Field in Normal State and Septic Shock

**DOI:** 10.3390/jcm14072496

**Published:** 2025-04-06

**Authors:** Athanasios Chalkias

**Affiliations:** 1 Institute for Translational Medicine and Therapeutics, University of Pennsylvania Perelman School of Medicine, Philadelphia, PA 19104-5158, USA; thanoschalkias@yahoo.gr; 2 OUTCOMES RESEARCH Consortium^®^, Houston, TX 77030, USA; 3 Department of Critical Care Medicine, General Hospital of Piraeus “Tzaneio”, 18536 Piraeus, Greece

**Keywords:** septic shock, microcirculation, magnetic field, cardiovascular dynamics, circulatory dynamics, hemodynamics, translational physiology, applied physiology, critical care medicine, anesthesiology

## Abstract

**Background**: Capillary tortuosity is a morphological variant of microcirculation. However, the mechanisms by which tortuous vessels meet metabolic requirements in health and disease remain unknown. We recently reported that capillary tortuosity score (CTS) is significantly higher in patients with septic shock than in steady-state individuals, and that CTS is significantly associated with alveolar-to-arterial oxygen (A-a O_2_) gradient and oxygen debt in septic shock patients. **Objective**: We aimed to investigate the characteristics of the magnetic fields in the sublingual microcirculation of individuals with normal physiology and patients with septic shock. **Methods**: Systemic hemodynamics were recorded, and sublingual microcirculation was monitored using sidestream dark field (SDF+) imaging. The number of capillary red blood cells (N_RBC_), the intensity of the magnetic field of a red blood cell (H_RBC_), the intensity of the magnetic field of each capillary (H_CAP_), and the intensity with which the magnetic field of a capillary acts on an RBC (F_CAP_) were calculated. **Results**: Significant differences in macro- and microhemodynamic variables were observed between the two groups. Although N_RBC_ was significantly higher in individuals with steady-state physiology [87.4 (87.12) vs. 12.23 (6.9)], H_RBC_ was significantly stronger in patients with septic shock [5.9 × 10^−16^ (6.9 × 10^−16^) A m^−1^ vs. 1.6 × 10^−15^ (1.4 × 10^−15^) A m^−1^]. No significant difference was observed in H_CAP_ [2.16 × 10^−14^ (2.17 × 10^−14^) A m^−1^ vs. 1.34 × 10^−14^ (1.23 × 10^−14^) A m^−1^] and F_CAP_ [1.66 × 10^−24^ (3.36 × 10^−24^) A m^−1^ vs. 6.44 × 10^−25^ (1.1 × 10^−24^) A m^−1^] between the two groups. In patients with septic shock, H_RBC_ was associated with De Backer score (rho = −0.608) and venous–arterial carbon dioxide difference (rho = 0.569). In the same group, H_CAP_ was associated with convective oxygen flow (rho = 0.790) and oxygen extraction ratio (rho = −0.596). Also, F_CAP_ was significantly associated with base deficit (rho = 0.701), A-a O_2_ gradient (rho = 0.658), and oxygen debt (rho = −0.769). **Conclusions**: Despite the microcirculatory impairment in patients with septic shock, H_RBC_ was significantly stronger in that group than in steady-state individuals. Also, H_CAP_ and F_CAP_ were comparable between the two groups. Tortuous vessels may function as biomagnetic coils that amplify RBC-induced magnetic fields, enhancing perfusion and oxygenation of adjacent tissues.

## 1. Introduction

Adequate microvascular perfusion is fundamental for maintaining cellular homeostasis and viability. Perfusion is probably more important than local oxygen concentration as hypoxia does not equate to specific oxygen levels. The latter vary widely in tissues [1,2,3], with most of them functioning physiologically at levels equivalent to an atmosphere of 0.5–9% of oxygen [2,4,5]. This well-compensated state of low partial pressure of oxygen is the result of the integrated activity of all organ systems and the fine communication among biological levels that maintain normal hemodynamic coherence over a range of perfusion and/or oxygen concentration changes. Intriguingly, critically ill patients have an even more complicated response and adaptation to abnormal alterations as their compensatory mechanisms strive to cover metabolic oxygen demands.

Capillary tortuosity is a morphological variant of microcirculation of various species and tissues [6,7,8,9]. This structural remodeling is observed in a variety of normal and pathological processes that share a common feature, namely a change in blood distribution and oxygen transport to tissue. In addition, tortuous vessels have distinct morphology (e.g., twisting or looping) and properties compared to normal linear vessels. For instance, they are often excessively permeable, while their endothelial cells differ from those of linear vessels [9,10]. The mechanisms by which torturous vessels meet metabolic requirements cannot be elucidated by current theoretical and research approaches and remain unknown. Their investigation requires an integrative approach to study the interaction between organ systems and subsystems using principles of applied and natural sciences.

We recently reported that sublingual tortuosity is essentially absent in normal states and increases significantly in severe sepsis and septic shock, presumably as an adaptive response to sepsis-induced alterations and disequilibrium in the demand, supply, and extraction of oxygen [11]. Close inspection and meticulous analysis of the morphology of tortuous vessels in our patients revealed a specific pattern and a shape that is similar to that of an electromagnetic coil, i.e., a spiral or helical electrical conductor generating a magnetic field. Of note, early studies on the magnetic properties and water dynamics of human red blood cells (RBCs) demonstrated the existence and dependence of their local magnetic fields, reflected by the chemical shifts and relaxation rates of the observed nuclei, cell density, and the magnetic state of hemoglobin [12]. This magnetic field is generated by the charges located on the RBC membrane and can influence the processes occurring both outside the erythrocyte and inside it [13].

According to the author’s perspective, tortuous vessels act as microvascular coils that produce a magnetic field induced by the flow of RBCs, enhancing the perfusion and oxygenation of adjacent tissues. This study investigated the characteristics of the magnetic fields in the sublingual microcirculation of individuals with normal physiology and patients with septic shock.

## 2. Materials and Methods

Twenty ASA 1 individuals with steady-state physiology and 13 patients with septic shock [mean SOFA score: 11 (2.7)] were analyzed [11]. The underlying studies were conducted in accordance with Good Clinical Practice guidelines, the Declaration of Helsinki, and relevant regulatory requirements. The original protocol (NCT03851965) was approved by the UHL Institutional Review Board (IRB no. 60580, 11 December 2018). Informed consent was obtained from all subjects involved in the study or their next-of-kin. This work is reported according to STROCSS criteria [14].

### 2.1. Study Objective

The primary objective was to analyze the intensity of the magnetic fields in the sublingual microcirculation of individuals with normal state and in patients with septic shock.

### 2.2. Patients and Measurements

The detailed protocol has been described elsewhere [11]. Briefly, we considered adults undergoing elective major non-cardiac surgery and adults with septic shock requiring emergency abdominal surgery (File S1). General anesthesia was maintained by inhalation of desflurane, and the depth of anesthesia was adjusted to maintain a Bispectral Index (BIS, Covidien, France) value between 40 and 60. Normoxemia, normocapnia, normothermia, and normoglycemia were maintained during this study. The radial artery was cannulated and connected to a FloTrac/EV1000 clinical platform (Edwards Life Sciences, Irvine, CA, USA) to directly measure macrohemodynamics. The internal jugular vein was cannulated with a triple-lumen central venous catheter that was connected to a pressure transducer to measure central venous pressure and oxygen saturation.

Sublingual microcirculation was monitored using sidestream dark field (SDF+) imaging (Microscan; Microvision Medical BV, Amsterdam, The Netherlands), in accordance with the guidelines on the assessment of sublingual microcirculation of the European Society of Intensive Care Medicine [15,16]. In both groups, microcirculation was assessed 30 min after the induction of general anesthesia before surgical incision. In patients with septic shock, microcirculation was assessed after normalization of macrohemodynamics.

Prior to advancing the imaging probe towards the targeted microcirculatory area, excess secretions were gently removed using a sterilized gauze moistened with sterile water maintained at the same temperature as the patient’s sublingual area. The latter was measured with a common digital oral thermometer. After carefully advancing, positioning, and maintaining gentle contact of the MicroScan lens with the sublingual mucosa, we optimized video quality by optimizing focus, illumination, and contrast. Image stability free of motion, bubbles, and pressure-induced artifacts was achieved due to the patient’s neuromuscular blockade and the stable placement and immobilization of the examiner’s hand on the patient’s headrest. Microcirculation videos were obtained from at least five sites with the probe maintained on the same landmark for the entire duration of each recording. The process of video acquisition was further mediated by a validated automatic algorithm software [AVA 4.3C (Microvision Medical, Amsterdam, The Netherlands)] to ensure adequate brightness, focus, and stability.

Before sublingual perfusion analysis, all videos were evaluated by two experienced raters blinded to all patient data according to a modified microcirculation image quality score (MIQS) [16]. The best three videos from each recording were analyzed offline by a blinded investigator and with AVA4.3C research software [15,17]. We analyzed the De Backer score (in mm^−1^; this equals the number of crossings of the capillary web × 21), the consensus proportion of perfused vessels (consensus PPV; this is a ratio of perfused capillaries from all visible capillaries given as a percentage), the consensus PPV (small) (i.e., the PPV of vessels with a diameter ≤ 25 μm), and the microvascular flow index (MFI). To assess the latter, the screen is divided into four quadrants and a score between 0 and 3 reflecting the average RBC velocity is given per quadrant. Thereafter, MFI is calculated as the mean MFI averaged over the four quadrants.

Vessel diameter, vessel length, and RBC velocity were determined with the latest version of AVA software using a modified optical flow-based algorithm; the method uses per video frame data to measure the overall velocity per vessel segment. Sublingual tortuosity was assessed with the capillary tortuosity score (CTS), a microvascular score with a good inter- and intra-observer variability, that morphologically assesses microvascular architecture based on the number of twists per capillary existing in the field of view [18]. Capillary shear stress, oxygen transport, and hemodynamic/metabolic variables were assessed as previously described (Table S1) [11].

### 2.3. Intensity of the Magnetic Field

The intensity of the magnetic field (H) can be calculated only in capillaries, where the cells flow singly without tumbling motion [19]. For this purpose, the number of capillary RBCs (N_RBC_) was estimated according to the rule of three, i.e., the hemoglobin value is equal to three times the RBC count and the hematocrit value is equal to three times the hemoglobin value [20]. Determination of hemoglobin concentration was also based on the volumetric relationship between the RBCs and the plasma, when blood is streaming through microvessels of different diameters (Table 1).

The distribution of RBCs and the velocity with which they move through capillaries depend upon the forces influencing blood flow, the shape of circulatory channel, the size and mechanical properties of blood cells, and plasma viscosity. On the RBC membrane, there is a finite number of discrete charges, and each of these charges at some pre-selected point generates a magnetic field [19,24,25,26,27]. The intensity of the magnetic field of an RBC (H_RBC_) can be calculated by the following equation (SI system):

(1) $$H_{\mathrm{RBC}}=\frac{qV\sin\alpha }{4\pi R^{2}}\;\mathrm{ampere}\;\mathrm{per}\;\mathrm{meter}\;(A\;m^{-1})$$

where q is the RBC charge (Q_RBC_ = 3.2 × 10^−12^ C), V is the velocity of RBC in μm s^−1^, R is the distance of RBC motion, and α is the angle between the radius vector R and the direction of the V [19,24,25,26,27]. In this equation, R was set at a distance equal to the length of the microvessel, and angle α was set at 90° (sin α = sin 90° = 1).

The intensity of the magnetic field of several charges (RBCs) at some point in space is defined as the vector sum of the strengths generated by the charges at this point [24,25,26]. The intensity of the magnetic field of each capillary (H_CAP_) can be calculated by multiplying Equation (1) with N_RBC_ in the entire capillary [19,24,25,26,27]:

(2) $$H_{\mathrm{CAP}}=\frac{qV\sin\alpha }{4\pi R^{2}}\;\times \;N_{\mathrm{RBC}}\;(A\;m^{-1})$$

The intensity with which the magnetic field of a capillary (F_CAP_) acts on flowing RBCs can be calculated by the following equation [19,24,25,26,27]:

(3) $$F_{\mathrm{CAP}}=q\;H_{\mathrm{CAP}}\;V\;\sin\alpha \;(A\;m^{-1})$$

### 2.4. Statistical Analysis

Statistical analysis was performed using R v4.4 software. Descriptive statistics are presented as the mean (standard deviation; SD). The Mann–Whitney test with Cohen’s D was employed to assess the statistical significance of the differences in magnetic fields and other variables between groups. Spearman’s method was used to estimate the strength of the correlations between N_RBC_, H_RBC_, and F_CAP_ and the various macro/microcirculatory, perfusion, and oxygen transport variables. In order to adjust for the presence of multiple comparisons, the Benjamini–Hochberg false discovery rate correction was utilized. *p*-values less than 0.05 were considered significant.

## 3. Results

### 3.1. Demographics, Systemic Hemodynamics, and Microcirculation

A statistically significant difference was observed in age [39.7 (7.06) vs. 70.5 (8.97), *p* < 0.001], sex [male: 12 (60%) vs. 9 (69.2%), *p* = 0.009], and body mass index [24.5 (1.52) vs. 27.15 (2.22), *p* = 0.002], but not in body surface area [1.93 (0.13) vs. 1.96 (0.1), *p* = 0.396] or temperature [36.72 (0.12) vs. 36.72 (0.49) °C, *p* = 0.823] between the two groups [11].

Medical history was unremarkable in individuals with steady-state physiology. In patients with septic shock, medical history revealed ischemic heart disease [5 (38.5%)], hypertension [10 (76.9%)], hypercholesterolemia [8 (61.5%)], diabetes [6 (46.2%)], stroke [3 (23.1%)], and chronic obstructive pulmonary disease [4 (30.8%)] [11].

**Figure 1:**
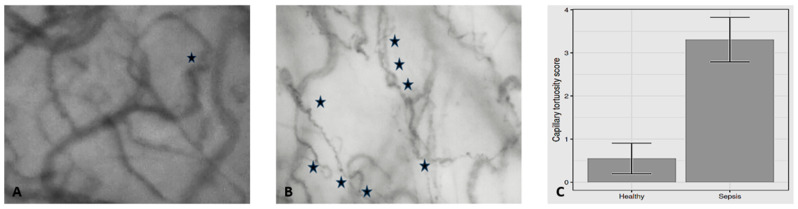
Differences in capillary tortuosity score between steady-state (**A**) and septic shock (**B**). Mean (SD) capillary tortuosity score (**C**) was 0.55 (0.76) vs. 3.31 (0.86), respectively (*p* < 0.001). Each dark blue star indicates the presence of twist(s) in a capillary. Adapted from Reference [11].

**Figure 2:**
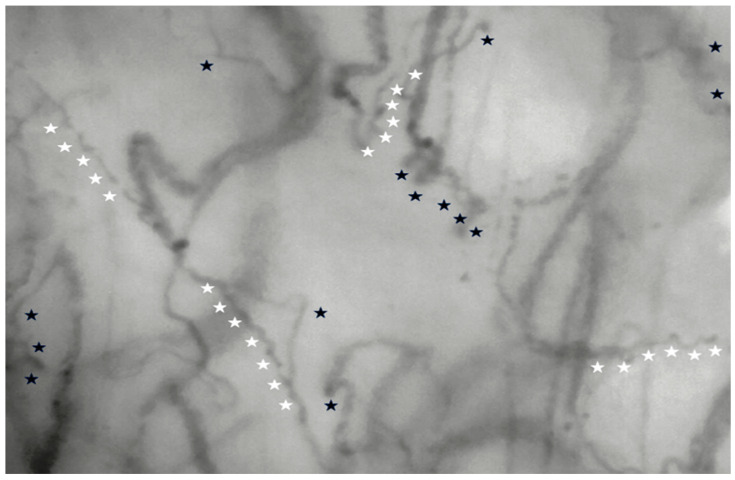
Increased capillary tortuosity in septic shock. Each dark blue star indicates the presence of twist(s) in a capillary. White stars indicate spiral-shaped vessels characterized by a specific pattern of helical twists (microvascular or biomagnetic coils).

Differences in macro- and microhemodynamic variables are presented in Table 2 and Table 3, respectively. Oxygen transport and metabolic variables are presented in Table 4. Capillary tortuosity score was significantly higher [0.55 (0.76) vs. 3.31 (0.86), *p* < 0.001] and associated with alveolar-to-arterial oxygen gradient (A-a O_2_ gradient) (r = 0.658, *p* = 0.015) and oxygen debt (r = −0.769, *p* = 0.002) in patients with septic shock (Figure 1 and Figure 2) [11].

### 3.2. Magnetic Field Parameters

The N_RBC_ was significantly higher in individuals with steady-state physiology compared to patients with septic shock [87.4 (87.12) vs. 12.23 (6.9), *p* < 0.001, Cohen’s D = 1.37]. However, H_RBC_ was significantly stronger in patients with septic shock [5.9 × 10^−16^ (6.9 × 10^−16^) A m^−1^ vs. 1.6 × 10^−15^ (1.4 × 10^−15^) A m^−1^, *p* = 0.009, Cohen’s D = 0.94]. No significant difference was observed in H_CAP_ [2.16 × 10^−14^ (2.17 × 10^−14^) A m^−1^ vs. 1.34 × 10^−14^ (1.23 × 10^−14^) A m^−1^, *p* = 0.203, Cohen’s D = 0.44] and F_CAP_ [1.66 × 10^−24^ (3.36 × 10^−24^) A m^−1^ vs. 6.44 × 10^−25^ (1.1 × 10^−24^) A m^−1^, *p* = 0.167, Cohen’s D = 0.36] between the steady-state and septic shock patients.

In steady-state individuals (intact hemodynamic coherence), the N_RBC_ was significantly associated with H_CAP_ (rho = 0.640, *p* = 0.002, adjusted *p* = 0.029) and convective oxygen flow (rho = 0.689, *p* < 0.001). The H_RBC_ was associated with H_CAP_ (rho = 0.683, *p* < 0.001, adjusted *p* = 0.017) and microvascular wall shear stress (rho = 0.569, *p* = 0.008). The H_CAP_ was significantly associated with De Backer score (rho = 0.739, *p* < 0.001, adjusted *p* = 0.003), microvascular wall shear stress (rho = 0.545, *p* = 0.01), venous–arterial carbon dioxide difference (rho = 0.532, *p* = 0.015), and convective oxygen flow (rho = 0.638, *p* = 0.002). Also, F_CAP_ was significantly associated with consensus PPV (small) (rho = −0.458, *p* = 0.04), diastolic arterial pressure (rho = −0.471, *p* = 0.036), base deficit (rho = −0.463, *p* = 0.04), expected A-a O_2_ gradient for age (rho = −0.684, *p* < 0.001), and arterial oxygen content (rho = −0.474, *p* = 0.034).

In patients with septic shock, the N_RBC_ was significantly associated with H_RBC_ (rho = −0.718, *p* = 0.005). The H_RBC_ was significantly associated with De Backer score (rho = −0.608, *p* = 0.02) and venous–arterial carbon dioxide difference (rho = 0.569, *p* = 0.04). A marginal association was observed between H_RBC_ and H_CAP_ (rho = 0.549, *p* = 0.05). The H_CAP_ was significantly associated with convective oxygen flow (rho = 0.790, *p* = 0.001) and O_2_ER (rho = −0.596, *p* = 0.03). Also, F_CAP_ was significantly associated with base deficit (rho = 0.701, *p* = 0.007), A-a O_2_ gradient (rho = 0.658, *p* = 0.014), and oxygen debt (rho = −0.769, *p* = 0.002).

## 4. Discussion

The present analysis revealed that although N_RBC_ was significantly higher in steady-state individuals, H_RBC_ was significantly stronger in patients with septic shock while H_CAP_ and F_CAP_ were comparable between the two groups. Several associations were observed—as expected—in individuals with steady-state physiology and intact hemodynamic coherence. In septic patients, N_RBC_ was negatively associated with H_RBC_. The latter was negatively associated with De Backer score and positively associated with venous–arterial carbon dioxide difference. The magnetic field of each capillary (i.e., H_CAP_) was positively associated with convective oxygen flow and negatively associated with oxygen extraction ratio. The intensity of the capillary magnetic field (i.e., F_CAP_) was positively associated with A-a O_2_ gradient and base deficit and negatively associated with oxygen debt.

The pathophysiology of impaired oxygen transport and extraction in sepsis is complex and may include microvascular injury, abnormal distribution of blood flow, and increases in the diffusion gradient from capillaries to mitochondria [11]. In addition, the well-established association of lower levels of adenosine triphosphate with mortality [28,29,30,31,32] suggests that mitochondrial dysfunction is another cause of impaired oxygen utilization and organ failure in septic conditions [33,34,35]. We have previously characterized microvascular tortuosity as an adaptive and compensatory phenomenon that improves both convective delivery to the capillary bed and diffusive transport from RBCs to mitochondria [11]. The present analysis shows that these processes may be largely dependent on the increase in H_RBC_, H_CAP_, and F_CAP_ of tortuous microvessels and the associated enhancement of permeation across biological barriers (i.e., magnetophoresis) [36,37].

**Figure 3:**
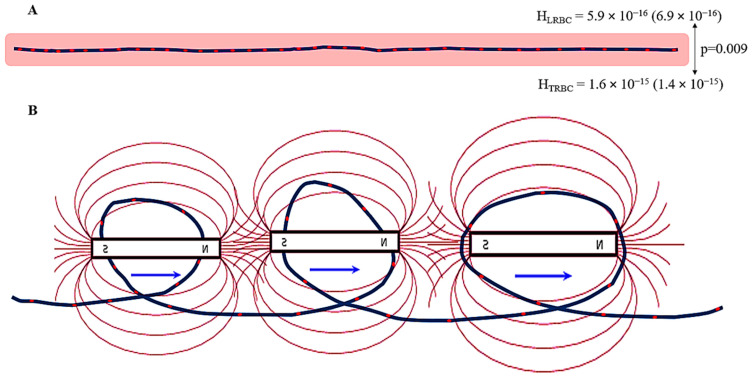
Motion of charged red blood cells (red dots) in linear (steady-state; **A**) and tortuous (septic shock; **B**) capillaries. Although the number of red blood cells is smaller in tortuous vessels, the magnetic field strength of the latter (H_TRBC_—circular areas) is significantly greater than that of linear vessels (H_LRBC_—pink area). In addition, the action of neighboring magnetic fields generated by adjacent twists (i.e., F_CAPs_) enhances the motion of red blood cells in tortuous vessels, thereby increasing their deformation index, facilitating their motion, and reinforcing H_TRBC_. These phenomena can improve gas exchange in systemic and pulmonary networks; promote aerobic glycolysis, oxidative phosphorylation, and adenosine triphosphate synthesis; and reduce bacterial growth in septic conditions. H_LRBC_ and H_TRBC_ are in A m^−1^.

Previous studies reported that the charges located on the RBC membrane generate a magnetic field (i.e., H_RBC_) which depends on cell density and the magnetic state of hemoglobin [12,19,24,27]. The electronegative surface charges and the ionic cloud that normally surrounds RBCs prevent them from approaching each other. Instead, these phenomena force RBCs to move in an oscillating pattern while maintaining a distance of 2–8 μm from each other [27]. The H_RBC_ can also affect the motion of other charged elements in blood, such as platelets and artificial magnetic particles used in the targeted transport of drugs, as well as various processes occurring inside the RBC [13,24]. Despite the seemingly critical role of RBC charges in capillary flow and cell homeostasis, the fact that N_RBC_ was significantly higher in steady-state individuals while H_RBC_ was significantly stronger in septic shock patients and that H_CAP_ and F_CAP_ were comparable between the two groups suggests the involvement of additional factors in the increase in these microvascular magnetic fields in sepsis. Notably, the present study revealed important associations in the septic group: (1) N_RBC_ was negatively associated with H_RBC_; (2) H_RBC_ was negatively associated with De Backer score and positively associated with venous–arterial carbon dioxide difference; (3) H_CAP_ was positively associated with convective oxygen flow and negatively associated with oxygen extraction ratio; (4) F_CAP_ was positively associated with base deficit and A-a O_2_ gradient; and (5) F_CAP_ was negatively associated with oxygen debt. Furthermore, the association between H_RBC_ and H_CAP_ is plausible and supported by a strong physiological basis, while its Spearman’s rank correlation coefficient is indicative of a high/very high correlation regardless of the borderline *p*-value. These findings together with the results of the previous report from our research group [11] strongly suggest increased capillary tortuosity as a major cause of increased H_RBC_, H_CAP_, and F_CAP_ in septic conditions (Figure 3).

Similarly to the author’s own opinion, i.e., that tortuous vessels are active microvascular (biological) magnetic coils that enhance perfusion and oxygenation of adjacent tissues, other authors have also characterized blood vessels as active electric circuits that influence nearby cells and tissues [19]. We know from physics that the strength of a magnetic field can be modified by changing the amount of current flowing a coil. In a similar manner, the tortuosity-mediated increase in H_RBC_, H_CAP_, and F_CAP_ may maintain or enhance cell metabolism and homeostasis [11,38,39,40,41]. Also, the existence of regions of different magnetic susceptibility in RBCs induces intrinsic non-linear magnetic field gradients when these cells are under the action of neighboring magnetic fields (i.e., F_CAP_) generated by adjacent twists [36,42,43]. These F_CAPs_ further enhance the oscillatory motion of RBCs, reinforce H_RBC_ and H_CAP_, and increase the deformation index of RBCs, facilitating their motion in tortuous capillaries [43]. These phenomena can improve gas exchange in systemic and pulmonary networks and alter the equilibrium of electric potentials around the inner mitochondrial membrane, promoting aerobic glycolysis, oxidative phosphorylation, and adenosine triphosphate synthesis [19,44,45,46,47,48]. Furthermore, the reinforced H_RBC_, H_CAP_, and F_CAP_ may affect crucial physicochemical processes in Gram-positive and Gram-negative bacteria, thereby reducing bacterial growth [49].

Previous studies have shown that local exposure to static magnetic fields can increase microcirculatory blood flow and blood velocity by 20–45%, with the greatest increase observed after the end of the exposure period [50,51,52,53]. In another study with rabbits subjected to norepinephrine-induced vasoconstriction, the exposure to static magnetic field led to increased vasomotion and vasodilation of cutaneous microvasculature [54,55]. In contrast, in reserpine-treated (vasodilation) rats with hypotension and depletion of catecholamine reserves, a 12-week exposure to static magnetic field significantly reduced hypotension and restored microvascular tone [56,57,58]. Similar findings were reported in other studies assessing the effects of pulsed electromagnetic fields on microcirculation [50]. The characteristics of tortuous vessels (constant RBC flow, coil-shaped morphology) suggest that their Hs exert a homeostatic action by combining the action of both static magnetic and pulsed magnetic fields. Therefore, it is reasonable to assume that H_RBC_, H_CAP_, and F_CAP_ improve the mechanical properties of the endothelial membrane through modifications of cytoskeletal actin filaments, thereby improving endothelium membrane compliance, mechanoreceptor sensitivity to shear stress, nitric oxide release, and vasomotion [59,60,61].

According to Equations (1) and (2), H_RBC_ and H_CAP_ are proportional to V and inversely proportional to R. Although V was not significantly different between the two groups, R (vessel length) was significantly shorter in patients with septic shock. To the best of the author’s knowledge, the structural length of each capillary is not reduced per se in septic shock. Therefore, the decrease in functional vessel density in septic patients may not only indicate a shut-down of vulnerable microcirculatory units but, also, a prudent and strategic distribution of blood flow from the longer to the shorter capillaries in order to increase local H_RBC_ and H_CAP_. Therefore, it is reasonable to argue that (1) seemingly harmful changes in the microcirculation are not always detrimental to tissue perfusion and oxygenation (i.e., hemodynamic coherence); (2) we should not treat these disorders blindly in all patients; and (3) increased capillary tortuosity is an active process of adaptation to conditions characterized by circulatory failure and increased oxygen demand that must be strongly supported.

We acknowledge some limitations. Although the present physiological study includes a relatively small sample size, data collection and analyses were conducted by blinded investigators, minimizing inter-observer bias and increasing the credibility of study results and conclusions. Of note, Cohen’s Ds and Spearman’s rank correlation coefficients are indicative of large effect sizes and high/very high correlations, respectively, regardless of the *p*-values. In addition, at each measurement point, we recorded sublingual microcirculation videos from at least five sites and followed the guidelines on the assessment of sublingual microcirculation of the European Society of Intensive Care Medicine [15]. We also maintained normoxia, normocapnia, normoglycemia, and normothermia to minimize the iatrogenic effects on microvascular perfusion [1,62,63,64]. In the equations used in the present analysis, the angle α was set at 90°. In other angles, the strength of H_RBC_, H_CAP_, and F_CAP_ may be slightly different. Despite these limitations, the findings of the present study provide important insights that significantly deepen our knowledge on the role of capillary tortuosity in conditions characterized by circulatory failure and an increase in oxygen demand.

## 5. Conclusions

Although N_RBC_ was higher in steady-state individuals, H_RBC_ was significantly stronger in patients with septic shock while H_CAP_ and F_CAP_ were comparable between the two groups. The results of the present analysis together with our previous report [11] demonstrate for the first time that capillary tortuosity underlies increased microvascular H_RBC_, H_CAP_, and F_CAP_ in septic conditions. These findings provide an exciting new avenue for further investigation and highlight the importance of the emerging interdisciplinary field of quantum biology in critical care medicine and physiology.

## 6. Future Perspectives

Sepsis is a life-threatening condition that arises from a dysregulated systemic inflammatory response to infection. Its impact on the cardiovascular system manifests as hypotension, abnormal microvascular flow patterns, and disruptions in capillary blood flow, which impair oxygen delivery to organs with elevated metabolic demand [65,66,67,68]. This microcirculatory dysfunction contributes significantly to morbidity and mortality. While systemic hemodynamic parameters are routinely monitored and targeted in treatment, the intricate dynamics within the microvasculature often remain obscured.

Red blood cells are fundamentally magnetic due to the magnetic properties of hemoglobin. Even in the absence of an external magnetic field, the collective orientation and flow of RBCs within the microvasculature generate localized magnetic fields. The present study has highlighted the role of H_RBC_, H_CAP_, and F_CAP_ within the microvasculature as a critical factor influencing tissue perfusion and oxygenation, particularly in the context of septic shock. While the findings reported in this article place research on microvascular magnetic fields in septic shock in its nascent stage, the potential for future developments is significant. Areas for exploration that could revolutionize our understanding of H_RBC_, H_CAP_, and F_CAP_ include, but are not limited to, the following:

- Development of novel microvascular magnetic field monitoring techniques: The clinical introduction of hand-held microscopes allows clear visualization of the microcirculation and flowing RBCs. The promising results of the present study together with future technological advances could lead to the development of fully automated software for the analysis of H_RBC_, H_CAP_, and F_CAP_ in patients with septic shock.
- Investigating the association between microvascular magnetic fields and microvascular (dys)function: More research is needed to establish a clear association between the characteristics of RBC- and tortuosity-induced magnetic fields and key hemodynamic/microvascular/metabolic/oxygen transport parameters, such as RBC velocity, capillary perfusion, and oxygen extraction. Understanding these relationships is crucial for developing targeted therapies.
- Exploring the therapeutic potential of external magnetic fields: The present study evaluated H_RBC_, H_CAP_, and F_CAP_ in normal state and septic shock. Investigating the application of specific parameters, such as external magnetic fields, to modulate microvascular magnetic fields and improve tissue perfusion and oxygenation of adjacent tissues could offer a novel therapeutic approach in critically ill patients.
- Overcoming the limitations of current models: Accurately modeling the complex interplay between perfusion, microvascular magnetic field generation, and oxygenation requires further refinement of existing or novel translational/integrative/computational models.
- Targeting microvascular magnetic field influencing factors: Developing therapies that specifically address factors that disrupt microvascular magnetic fields, such as inflammation, endothelial dysfunction, and coagulation abnormalities, could indirectly optimize H_RBC_, H_CAP_, and F_CAP_ behavior and hemodynamic coherence.
- Personalized medicine approach: Recognizing the heterogeneity of septic shock, a personalized approach that considers individual differences in capillary tortuosity, and H_RBC_, H_CAP_, and F_CAP_ characteristics could lead to more effective and targeted therapies. For example, monitoring the H_RBC_, H_CAP_, and F_CAP_ response to different drug concentrations or fluid volumes could help determine the optimal dosage for each patient, allowing for a timely individualized treatment strategy and improving outcomes.

The study of microvascular magnetic fields represents a highly innovative and potentially transformative area of research. While further investigation at various integrative levels is necessary to fully elucidate the complexities of H_RBC_, H_CAP_, and F_CAP_, the potential applications are immense for both healthy individuals and critically ill patients. Understanding and manipulating these magnetic fields could offer novel therapeutic avenues for improving tissue perfusion and oxygenation and, ultimately, patient outcomes.

## Figures and Tables

**Table 1:** Reduction in the hematocrit by reducing the microvessel diameter.

Microvessel Diameter (μm)	Blood Volume		Average Velocity of RBCs *
	Microvessel Hct (%)	Plasma (%) (1 − Microvessel Hct)	
1100	40.5	59.5	100
750	40.1	59.9	101
450	39.8	60.2	103
250	39.2	60.8	106
95	33.6	66.4	135
50	28.0	72.0	175

* That of plasma = 100. Hct, hematocrit; RBC, red blood cell. The table gives the change in the volumetric relationship between the RBCs and the plasma, when blood from a healthy person is streaming through microvessels of different diameters, and the calculated average velocities of the RBCs in proportion to those of the plasma. With decreasing diameter of the microvessels below 0.1 mm, the relative RBC volume very rapidly decreases, while the velocity of the RBCs in proportion to that of the plasma very rapidly increases. Adapted from References [21,22,23].

**Table 2:** Differences in macrohemodynamic variables between groups.

	Steady-State (n = 20)	Septic Shock (n = 13)	*p*-Value	Cohen’s D
Heart rate (bpm)	67.5 (6.98)	96.92 (23.5)	<0.001	1.89
Systolic arterial pressure (mmHg)	120 (7.43)	116.92 (22.13)	0.863	0.21
Diastolic arterial pressure (mmHg)	71.25 (7.41)	66.54 (14.91)	0.595	0.43
Mean arterial pressure (mmHg)	88.13 (6.97)	83.62 (16.76)	0.956	0.38
Cardiac output (L min^−1^)	5.04 (0.68)	5.48 (1.01)	0.16	0.55
Cardiac index (L min^−1^ m^−2^)	2.6 (0.3)	2.75 (0.54)	0.554	0.36
Stroke volume (mL beat^−1^)	74.7 (9.57)	61.54 (26.04)	0.001	0.74
Stroke volume variation (%)	5.9 (1.83)	12.69 (5.14)	<0.001	1.94
Systemic vascular resistance (dynes s cm^−5^)	1306.3 (176.32)	896.31 (247.49)	<0.001	1.98
Central venous pressure (mmHg)	7.05 (0.69)	11.31 (4.29)	<0.001	1.56
Mean circulatory filling pressure analogue (mmHg)	13.06 (0.86)	18.62 (4.61)	<0.001	1.89
Cardiac power output (W)	0.99 (0.17)	1.01 (0.27)	0.54	0.12
Power (W)	0.9 (0.16)	0.87 (0.24)	0.696	0.36

Adapted from Reference [11].

**Table 3:** Differences in microhemodynamic variables between groups.

	Steady-State (n = 20)	Septic Shock (n = 13)	*p*-Value	Cohen’s D
De Backer score (mm^−1^)	3.7 (1.17)	3.62 (1.19)	0.754	0.07
Consensus PPV (%)	94.15 (5.66)	60.2 (11.3)	<0.001	4.08
Consensus PPV (small) (%)	122.89 (146.74)	50.57 (12.64)	<0.001	3.51
Microvascular flow index (AUs)	2.76 (0.25)	1.83 (0.61)	<0.001	2.2
Vessel diameter (μm)	10.07 (5.02)	4.35 (1.83)	<0.001	3.39
Vessel length (μm)	141 (154.25)	42.54 (15.98)	<0.001	3.15
Red blood cell velocity (μm s^−1^)	15.69 (15.02)	13.46 (12.45)	0.519	0.25
Wall shear stress (dyne cm^−2^)	3.86 (2.68)	0.72 (0.36)	<0.001	1.49
Capillary tortuosity score	0.55 (0.76)	3.31 (0.86)	<0.001	3.46

PPV, proportion of perfused vessels; AUs = arbitrary units. Data are presented as mean (SD) unless stated otherwise. Adapted from Reference [11].

**Table 4:** Differences in oxygen transport and metabolic variables between groups.

	Steady-State (n = 20)	Septic Shock (n = 13)	*p*-Value	Cohen’s D
Fraction of inspired oxygen	0.31 (0.03)	0.49 (0.16)	<0.001	1.71
pH	7.39 (0.02)	7.32 (0.11)	0.052	1.06
Arterial partial pressure of oxygen (mmHg)	92.5 (5.12)	104.31 (38.73)	0.971	0.48
Arterial partial pressure of carbon dioxide (mmHg)	39.2 (1.28)	36.85 (6.44)	0.64	0.57
Venous–arterial carbon dioxide difference (mmHg)	2.8 (0.89)	9 (1.87)	<0.001	4.56
Bicarbonate (mmol L^−1^)	25.6 (0.99)	21.19 (6.85)	0.003	1.02
Base deficit (mmol L^−1^)	2.08 (0.19)	−0.19 (7.59)	0.183	0.48
Hemoglobin (g dL^−1^)	14.06 (0.94)	9.73 (1.83)	<0.001	3.2
Lactate (mmol L^−1^)	0.81 (0.15)	3.45 (2.78)	<0.001	1.52
A-a O_2_ gradient (mmHg)	80.33 (25.43)	198.46 (126.17)	<0.001	1.46
Expected A-a O_2_ gradient for age (mmHg)	13.95 (1.77)	21.65 (2.24)	<0.001	3.92
Peripheral oxygen saturation (%)	99.6 (0.5)	95.31 (4.01)	0.001	1.7
Arterial oxygen saturation (%)	100 (0)	96.77 (3.11)	<0.001	1.67
Central venous oxygen saturation (%)	74.15 (2.3)	77.92 (6.12)	0.015	0.9
Oxygen extraction ratio (%)	25.85 (2.3)	19.31 (5.59)	0.001	1.67
Arterial oxygen content (vol%)	19.7 (1.3)	13.3 (2.41)	<0.001	3.53
Venous oxygen content (vol%)	14.73 (1.4)	10.76 (2.27)	<0.001	2.22
Venous–arterial oxygen content difference (vol%)	4.96 (0.78)	2.54 (0.74)	<0.001	3.17
Oxygen delivery (mL min^−1^)	973.88 (116.23)	724.19 (160.4)	<0.001	1.85
Oxygen consumption (mL min^−1^)	247.43 (35.64)	136.45 (41.54)	<0.001	2.92
Convective oxygen flow (μm^2^ sec^−1^ kg^−1^)	26.44 (39.96)	1.1 (1.41)	<0.001	0.81
Oxygen debt	−8.62 (1.13)	13.23 (29.62)	0.002	1.18

A-a, alveolar-to-arterial. Data are presented as mean (SD) unless stated otherwise. Adapted from Reference [11].

## Data Availability

Data will be made available upon request after publication through a collaborative process. Researchers should provide a methodically sound proposal with specific objectives in an approval proposal. Please contact the corresponding author for additional information.

## Supplementary Materials

The following supporting information can be downloaded at https://www.mdpi.com/article/10.3390/jcm14072496/s1, Table S1: Equations of main hemodynamic and oxygen transport parameters used in this study; File S1: Inclusion and exclusion criteria.

## Abbreviations

The following abbreviations are used in this manuscript:

RBC	Red blood cell
CTS	Capillary tortuosity score
H	Intensity of the magnetic field
N_RBC_	Number of capillary red blood cells
H_RBC_	Intensity of the magnetic field of a red blood cell
H_CAP_	Intensity of the magnetic field of each capillary
F_CAP_	Intensity of the capillary magnetic field acting on a single red blood cell
A-a O_2_ gradient	Alveolar-to-arterial oxygen gradient
