# Supplementary material for: On the Intensity of the Microvascular Magnetic Field in Normal State and Septic Shock
Source: J Clin Med. 2025 Apr 6;14(7):2496. doi: 10.3390/jcm14072496 (PMC11989563; doi:10.3390/jcm14072496)
Supplement: Supplementary file 1 [file jcm-14-02496-s001.zip › File S1.pdf]

Inclusion and Exclusion Criteria

Steady-state individuals

Consecutive adults who were scheduled to undergo elective major non-cardiac surgery with an expected duration of ≥2 h, under general anesthesia, were eligible for inclusion. Patients were American Society of Anesthesiologists physical status I. All operative approaches were eligible, including open and laparoscopic procedures. We excluded patients with any infection within the previous month; severe liver disease; need for renal replacement therapy; allergies; inflammatory or immune disorders; asthma; obesity (BMI ≥ 30 kg m^{-2}); mental disability or severe psychiatric disease; alcohol abuse; and connective tissue disease including rheumatoid arthritis, ankylosing spondylitis, and systemic lupus erythematosus. We also excluded patients who had previously received an organ transplant; who were treated with steroids, antipsychotic medication within the previous three months or with opioids during the past week; and who were involved in another study.

Patients with septic shock

We included consecutive adults fulfilling the following criteria: (a) American Society of Anesthesiologists' physical status I to V; and (b) septic shock requiring emergency abdominal surgery. Patients who were involved in another study were excluded.
