# Supplementary material for: On the Intensity of the Microvascular Magnetic Field in Normal State and Septic Shock
Source: J Clin Med. 2025 Apr 6;14(7):2496. doi: 10.3390/jcm14072496 (PMC11989563; doi:10.3390/jcm14072496)
Supplement: Supplementary file 1 [file jcm-14-02496-s001.zip › Table S1.pdf]

Table S1. Equations of main hemodynamic and oxygen transport parameters used in the study.

Mean circulatory filling pressure analogue	$Pmca = (a \times CVP) + (b \times MAP) + (c \times CO)$
Driving pressure for venous return	$VRdP = Pmca – CVP$
Resistance to venous return	$RVR = (Pmca – CVP) / CO$
Efficiency of the heart	$Eh = (Pmca – CVP) / Pmca$
Cardiac power	$Power = CO \times (MAP – CVP) \times 0.0022$
Cardiac power output	$CPO = (CO \times MAP) / 451$
Power efficiency	$Epower = \Delta((MAP\text{-}CVP) \times CO) \times 0.0022 / \Delta Pmca$
Volume efficiency	$Evol = \Delta(Pmca\text{-}CVP) / \Delta Pmca$
Arterial compliance	$C_{art} = SV / (SAP – DAP)$
Arterial resistance	$R_{art} = MAP / (SV \times HR)$
Venous compartment resistance	$R_{ven} = SVR \times 0.038$
Wall shear stress	$\tau_{w} = (\Delta P \times d) / 4L$
Oxygen extraction ratio	$O_2ER = VO_2 / DO_2 = (SaO_2 – ScvO_2) / SaO_2$
Arterial oxygen content	$CaO_2 = (0.0138 \times Hb \text{ x } SaO_2) + (0.0031 \times PaO_2)$
Venous oxygen content	$CvO_2 = (0.0138 \times Hb \times ScvO_2) + (0.0031 \times PcvO_2)$
Venous-arterial oxygen content difference	$CvO_2 – CaO_2$
Oxygen delivery	$DO_2 = CaO_2 \times CO \times 10$
Oxygen consumption	$VO_2 = C(a - v)O_2 \times CO \times 10$
Convective oxygen flow	$Q^{C}O_2 = \pi d^2/4 \times V_{RBC} \times [Hb] \times SO_2 \times C_{Hb}$
Oxygen debt	$OXD = 6.322 (Lactate) – 2.311 (BE) – 9.013$

Information is from reference 11.
